# The Challenges of Implementing Next Generation Sequencing Across a Large Healthcare System, and the Molecular Epidemiology and Antibiotic Susceptibilities of Carbapenemase-Producing Bacteria in the Healthcare System of the U.S. Department of Defense

**DOI:** 10.1371/journal.pone.0155770

**Published:** 2016-05-19

**Authors:** Emil Lesho, Robert Clifford, Fatma Onmus-Leone, Lakshmi Appalla, Erik Snesrud, Yoon Kwak, Ana Ong, Rosslyn Maybank, Paige Waterman, Patricia Rohrbeck, Michael Julius, Amanda Roth, Joshua Martinez, Lindsey Nielsen, Eric Steele, Patrick McGann, Mary Hinkle

**Affiliations:** 1 Multidrug-resistant organism Repository and Surveillance Network (MRSN)-Antimicrobial Resistance Monitoring and Research (ARMoR) Program, Walter Reed Army Institute of Research, Silver Spring, MD, United States of America; 2 Global Emerging Infectious Disease Surveillance and Response Section, Armed Forces Health Surveillance Section, Silver Spring, MD, United States of America; 3 Epidemiology and Analysis Section, Armed Forces Health Surveillance Center, Silver Spring, MD, United States of America; 4 Department of Laboratory Medicine, San Antonio Military Medical Center, San Antonio, TX, United States of America; Cornell University, UNITED STATES

## Abstract

**Objective:**

We sought to: 1) provide an overview of the genomic epidemiology of an extensive collection of carbapenemase-producing bacteria (CPB) collected in the U.S. Department of Defense health system; 2) increase awareness of the public availability of the sequences, isolates, and customized antimicrobial resistance database of that system; and 3) illustrate challenges and offer mitigations for implementing next generation sequencing (NGS) across large health systems.

**Design:**

Prospective surveillance and system-wide implementation of NGS.

**Setting:**

288-hospital healthcare network.

**Methods:**

All phenotypically carbapenem resistant bacteria underwent CarbaNP^®^ testing and PCR, followed by NGS. Commercial (Newbler and Geneious), on-line (ResFinder), and open-source software (Btrim, FLASh, Bowtie2, an Samtools) were used for assembly, SNP detection and clustering. Laboratory capacity, throughput, and response time were assessed.

**Results:**

From 2009 through 2015, 27,000 multidrug-resistant Gram-negative isolates were submitted. 225 contained carbapenemase-encoding genes (most commonly *bla*_KPC_, *bla*_NDM_, and *bla*_OXA23_). These were found in 15 species from 146 inpatients in 19 facilities. Genetically related CPB were found in more than one hospital. Other clusters or outbreaks were not clonal and involved genetically related plasmids, while some involved several unrelated plasmids. Relatedness depended on the clustering algorithm used. Transmission patterns of plasmids and other mobile genetic elements could not be determined without ultra-long read, single-molecule real-time sequencing. 80% of carbapenem-resistant phenotypes retained susceptibility to aminoglycosides, and 70% retained susceptibility to fluoroquinolones. However, among the CPB-confirmed genotypes, fewer than 25% retained susceptibility to aminoglycosides or fluoroquinolones.

**Conclusion:**

Although NGS is increasingly acclaimed to revolutionize clinical practice, resource-constrained environments, large or geographically dispersed healthcare networks, and military or government-funded public health laboratories are likely to encounter constraints and challenges as they implement NGS across their health systems. These include lack of standardized definitions and quality control metrics, limitations of short-read sequencing, insufficient bandwidth, and the current limited availability of very expensive and scarcely available sequencing platforms. Possible solutions and mitigations are also proposed.

## Background

“We changed again, and yet again, and it was now too late and too far to go back”

— Charles Dickens, Great Expectations

As antibiotic resistant pathogens, especially carbapenemase-producing bacteria (CPB), pose urgent public health threats [1, 2], enhanced surveillance and characterization of these organisms becomes crucial. Furthermore, innovative tests for characterization of resistant bacteria and enhanced surveillance comprise two of the five main goals of the 2015 National Action Plan to Combat Antibiotic Resistant Bacteria [3]. Clinicians and infection preventionists increasingly rely on and expect next generation sequencing (NGS) to quickly provide the highest resolution data available; an approach referred to as genomic epidemiology [4]. The two overarching goals of our study were to: a) implement NGS across a large and geographically-dispersed health system and surveillance network [5] in order to rapidly produce more accurate, reliable, and actionable data for infection control and antibiotic stewardship efforts; and b) gain insight into the molecular epidemiology of CPB encountered by hospitals in our surveillance network. Three secondary objectives of this project were to: 1) maximize sequencing capacity; 2) minimize turn-around time (72 hours or less) for outbreak investigations; and 3) make isolates, associated sequence and antibiogram data, and the architecture of a customized, enterprise-grade database available to others.

Although NGS is increasingly acclaimed for outbreak investigations, most such reports using NGS have been smaller or single facility reports [4, 6]. Additionally, the fewer larger or seminal reports leveraged well-resourced genomics institutes and/or crowdsourcing [7–9]. Our report, however, provides an overview of the molecular epidemiology of one of the most extensive U.S. collections of CPB to date. (From a total of >27,000 multidrug-resistant Gram negative clinical isolates, 225 contained one or more carbapenemase- encoding genes. These were found in 15 species, from 146 patients in 19 facilities). Moreover, these data were generated by a clinical hospital referral laboratory, without access to such genomics institutes. The perspective and many of the constraints we faced would be analogous to, or typical of, a state public health laboratory or the referral laboratory of a multi-facility healthcare system. We contend that other larger organizations are likely to face these considerations and challenges as they implement enterprise-wide NGS-based surveillance. If these challenges are not addressed, they could constrain the broader clinical-epidemiologic application of this technology (especially at federal and state public health laboratories) for such pathogens [10]. We also suggest possible solutions or mitigating strategies.

## Methods and Setting

This was a performance improvement project authorized by a public health and infection prevention and control mandate by the U.S. Army Medical Department. The authors did not have access to identifiable patient information. The data were anonymized/de-identified prior to access and analysis. Bacterial isolates were assigned a unique barcode. Detailed surveillance, characterization and reporting methods used were previously described [5].

Briefly, when a hospital identifies a carbapenem resistant phenotype, the isolate is sent to the referral laboratory where CarbaNP^®^ testing is performed. All CarbaNP positive isolates undergo PCR for all of the most common alleles of the carbapenemase-encoding genes *bla*_KPC_,*bla*_IMP_ *bla*_NDM_, *bla*_OXA_, and *bla*_VIM_. Isolates then undergo whole genome sequencing on the Illumina NextSeq 500 and/or MisSeq (Illumina Inc, San Diego, CA) bench top platforms using library preparations and chemistries previously described [11]. Isolates from novel species or those that contain plasmids or other mobile genetic elements are further sequenced on the PacBio RS II (Pacific Biosciences, Menlo Park, CA). Raw sequences are assembled and analyzed on a semi-automated sequencing pipeline using commercial (Newbler (Roche, Branford, CT); Geneious (Biomatters, Auckland, NZ)), and open source software (Btrim, FLASh, BLAST, Bowtie2, and Samtools [12–15], and on line resources (ResFinder) [16] (https://cge.cbs.dtu.dk//services/Resfinder/)). Sequences are uploaded to the NCBI Pathogen homepage at: http://www.ncbi.nlm.nih.gov/pathogens/ under submitter: http://www.ncbi.nlm.nih.gov/pathogens/contributors/. The BioProject is: http://www.ncbi.nlm.nih.gov/bioproject/300270.

**Fig 1:**
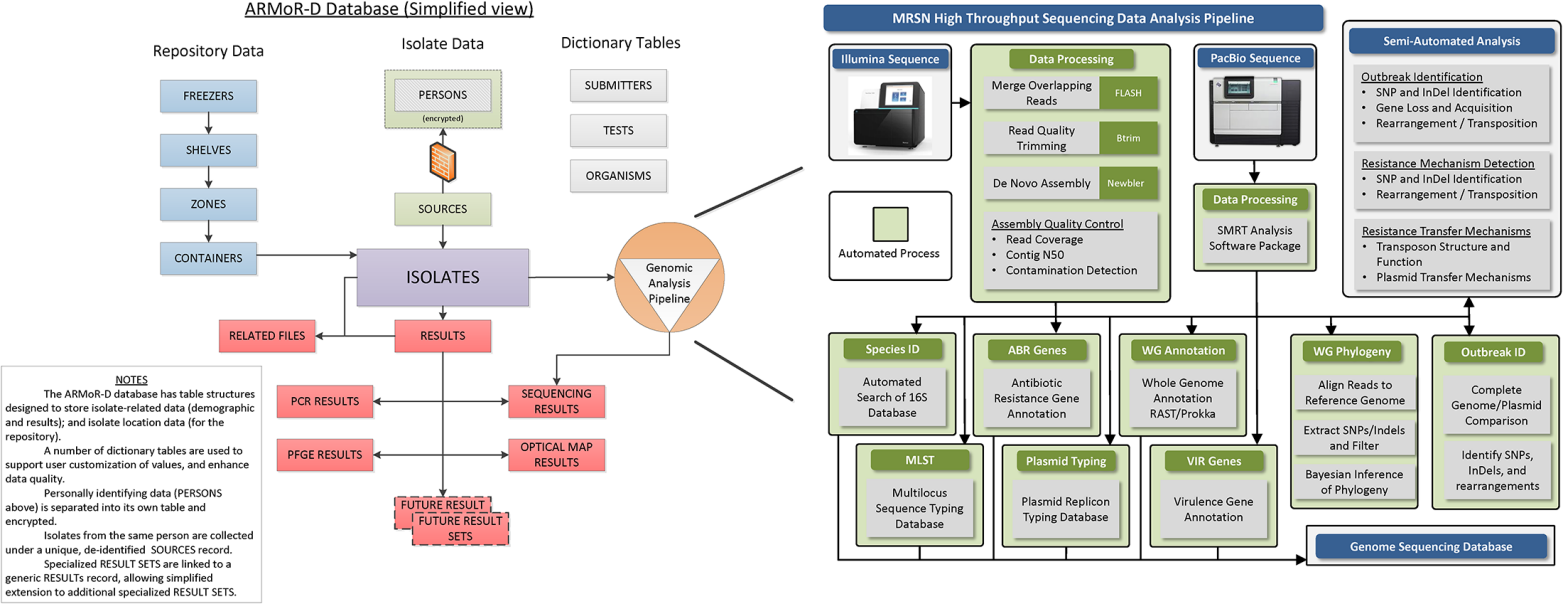
Customized Database for the ARMoR Program. The customized database of the Antimicrobial Resistance Monitoring and Research Program. The database contains secure personally identifiable information, sequence derived results, and phenotypic results, and manages isolate inventory in the repository. The database is also linked to a semi-automated bioinformatic pipeline.

The Department of Defense (DOD) system currently includes 288 fixed hospitals, up to six mobile hospitals in theaters of operation (Iraq, Afghanistan, etc.), and several military and civilian collaborating partners from 10 countries. Implementing NGS across that enterprise, we had three sub-tasks. The first was to maximize sequencing capacity for routinely sequencing every multidrug-resistant (MDR)-Gram negative, methicillin-resistant *Staphylococcus aureus*, vancomycin-resistant *Enterococcus*, and *Clostridium difficile* submitted to the repository (about 400 per month). The second was to minimize turn-around time (72 hours or less) for outbreak investigations. Given the number of hospitals and collaborators supported, it is conceivable that the center could be called upon to support two or three larger (12–15 patient/isolates) suspected outbreaks, and/or 5–7 smaller (3–5 patient/isolates) outbreaks simultaneously. The question that arose was whether two separate workflows or pipelines would be needed to accommodate both objectives. The third sub-task was to make isolates, associated sequence and antibiogram data, and the database architecture available to others. To accomplish the first two tasks, we use two automated liquid handlers for library preparation and a NextSeq 500 (Illumina, Inc, San Diego CA) for routine, higher volume projects that do not require longer reads. A MiSeq (Illumina, Inc, San Diego CA) is concurrently used for lower volume projects that require longer read-lengths. The automated liquid handlers minimize consumable, reagent, and labor costs in comparison to manual sample preparation. We also developed a sequence data analysis pipeline that performs the following functions in a semi-automated manner: 1) multi-locus sequence type (MLST) derivation; 2) genome-based phylogeny; and 3) identification of single nucleotide variations, genetic insertions and deletions, antibiotic resistance genes, and known virulence genes (Fig 1). The pipeline manages file naming and storage, ensures that basic data analyses are performed in a consistent and reproducible way, and generates quality control and results reports, thus freeing up bioinformaticians to apply their efforts to complex data analysis methods and the interpretation of results. Finally, since no commercial 'off-the-shelf' database exists for managing repository inventory, or linking clinical and demographic metadata to antibiotic susceptibility and sequence data, we developed our own (Fig 1).

## Results

### Overview of the Molecular Epidemiology of Carbapenemase-producing Bacteria in the DOD

From 2009 through 2015, ~35,000 clinical bacterial isolates were centralized tested and archived at the MRSN. Of those, ~27,000 were MDR Gram-negatives, of which ~1500 were phenotypically carbapenem-resistant. Of those, 225 were confirmed to contain one or more of *bla*_KPC_, *bla*_IMP_, *bla*_NDM_, *bla*_OXA48-like_, and *bla*_VIM_ genes based on CarbaNP testing, PCR, and NGS (Table 1). Gene content by PCR and NGS were concordant. These carbapenemase-encoding gene (CEG)-containing isolates were found in 15 different species and in 146 inpatients from 19 facilities. 84% (189) were infecting isolates, and the remainder were surveillance/colonizing isolates (Table 1). The isolates were collected from various sites: 31% urine, 23% wound, 21% respiratory, 17% blood, and 8% other. The most common mechanisms of carbapenem resistance were *bla*_KPC_ (66%) and *bla*_NDM_ (23%) in Enterobacteriaceae, and *bla*_OXA23-like_ variants in *Acinetobacter* spp. CEGs were very rarely found in *Pseudomonas aeruginosa*; Pseudomonads that were negative for CEGs likely encode mutations in OMP, efflux, or AmpC regulatory genes. *bla*_KPC_ (and bla_OXA23_ in *Acinetobacter* spp.) was the most prevalent mechanism in the contiguous U.S., while the other mechanisms were more commonly found in isolates originating outside the contiguous U.S.

**Table 1:** Overview of Carbapenemase-producing Genotypes in the Health and Surveillance Systems of the U.S. Department of Defense.

CarbapenemaseEncoding Gene	Species	# of Isolates Found In	# of Patients Found In	Infection by species	Surveillance by species	Facilities Found At
*bla*_KPC_	*K*. *pneumoniae*	117	71	93	24	A, B, C, E, F, K, U, N, Q, R
	*E*. *coli*	12	6	12	0	B, G N
	*E*. *cloacae*	3	3	3	0	B, D, N
	*P*. *aeruginosa*	10	2	10	0	N
	*K*. *oxytoca*	1	1	1	0	P
	*C*. *fruendii*	1	1	1	0	N
	*S*. *marcensens*	3	2	3	0	B, N
*bla*_NDM_	*K*. *pneumoniae*	18(3*)	9	14	4	H, M, N, P
	*E*. *cloacae*	3*	3	3	0	I, M, H
	*A*. *baumannii*	27 (3*)	24	27	0	F, I
	*A*. *schindleri*	1	1	0	1	N
	*P*. *stuartii*	2	1	2	0	H
	*E*. *coli*	3	3	2	1	H
	*C*. *freundii*	1	1	1	0	P
*bla*_IMP_	*P*. *aeruginosa*	1*	1	1	0	J
	*A*. *hemolyticus*	1*	1	1	0	J
	*A*. *junii*	2*	2	2	0	J
	*A*. *nosocomialis*	1*	1	0	1	J
	*A*. *baumannii*	1*	1	1	0	J
*bla*_VIM_	*C*. *freundii*	2	2	1	1	B, E
	*P*. *aeruginosa*	5	5	3	2	H, O, U, P, D
	*P*. *(new species)*	1	1	1	0	E
	*E*. *cloacae*	1	1	0	1	N
	*K*. *oxytoca*	2	1	1	1	P
*bla*_OXA48-like_	*E*. *coli*	5	1	5	0	N
	*M*. *morgannii*	1	1	1	0	N
TOTAL	*15 species*	225	146	189	36	19 facilities

^#^ = number of

There are two general categories of patients treated at overseas U.S. military hospitals, i) DOD healthcare beneficiaries (active duty, dependents, and retirees); and ii) non-U.S. coalition forces and host nation patients (in the contiguous U.S., only the former category is treated). Demographic and outcome data is consistently available only for the first category. Of the DOD healthcare beneficiaries, patients from whom a CEG-containing isolate was isolated were: 53% male; an average of 36.1 years old (range 0.5–76 years); with a mean of 9 diagnoses (range 2–30), and no infection-related deaths. However, there were reports of fatal outbreaks in host nation patients in some of the host nation hospitals that participated / submitted isolates to our network.

Detailed genomic epidemiology of these isolates is complicated with emerging and retreating clones or sub-populations depending on the species and gene, and is too large for a single manuscript, and beyond the scope of this report. Detailed of outbreaks or pseudo-outbreak reports have been provided to the individual or collectively involved facilities. In summary, genetically related CEG-containing isolates were found in more than one hospital, providing molecular evidence in support of nosocomial transmission via patient transfer to higher levels of care. Some occurrences or outbreaks of CEG-containing strains were clonal, but limited to a single facility, while others were not clonal either within or between facilities. Some occurrences and outbreaks of CEG-containing bacteria involved genetically related plasmids, and others involved several unrelated plasmids. De-duplicating on the basis of one isolate per patient per species per gene, the incidence of CEG-containing isolates peaked in 2013 (n = 65) and then decreased in 2014 (n = 22) remaining low through November 2015 (n = 8). However, based on the ratio of isolates confirmed to harbor a CEG (numerator) to the total number carbapenem-resistant phenotypes isolates tested with CarbaNP^®^ and/or PCR (denominator), the proportional prevalence was highest in 2013 (80/243; 32%), decreasing in 2014 to 18% (62/339), then increasing to 25% (43/175) in 2015. During this reporting period, the monthly average number of multidrug-resistant isolates submitted to the network has remained stable (~300) or slightly increased, while the intensity or consistency of surveillance has improved. No CEG-containing isolates were detected in the Northwest or Southwest regions of the DOD health system.

### Treatment Implications- Phenotype vs. Genotype

The most active antimicrobial agents against the CEG-carrying isolates were tigecycline (69% susceptible) and colistin. The least active agents were sulbactam (2% susceptible) and piperacillin-tazobactam (2.4% susceptible). Using provisional susceptibility breakpoints of </ = 4 for arbekacin and <2 for colistin, 44% and 94% of the CEG-containing isolates were susceptible, respectively. At a minimum inhibitory concentration (MIC) of 16, 71% of CPB were susceptible to arbekacin. These findings also illustrate the incremental value that mechanistic determinations add to the phenotypic antibiogram (MIC alone) for empiric treatment of these pathogens. For example, we compared the antibiograms of CEG-carrying isolates to the antibiograms of a similar-sized group of the same species that were phenotypically carbapenem resistant, but that were CEG-negative based on PCR or NGS. Based on the antibiograms of the carbapenem resistant phenotypes, 80% of CRE retained susceptibility to aminoglycosides, and 70% retained susceptibility to fluoroquinolones. However, among the organism confirmed to contain a CEG by genotype, fewer than 25% retained susceptibility to aminoglycosides or fluoroquinolones.

### Capacity and Turnaround Time

The sequencing pipeline provides a throughput for the above functions and annotations on 25 genomes per week, with a proven surge capacity of ten times that. A separate work flow for outbreak and routine is no longer needed. Additionally, by manipulating the chemistries of the library preparation kits size with computer processor time, we further reduced analytical turnaround time such that a SNP-based dendrogram and drug-resistance gene content report can be consistently provided to the submitting facility within 48–72 hours after we receive the specimens (11).

### Availability of Sequence Data, Isolates, and Database Structure

The third sub-task was to make isolates, associated sequence and antibiogram data, and the database architecture available to others. To accomplish this, we continue to upload sequence data to three public databases -GenBank, the NCBI's Genome Tracker & Pathogen Detection System, and the nascent interagency MDR-sequence database. Additionally, 'pathogen panels' consisting of 50 isolates of each species of MDR-ESKAPE organisms selected on the basis of genetic diversity (Pulsed Field Gel Electrophoresis (PFGE)/MLST) and anatomic site (10–15 each from blood, urine, wound, respiratory, and surveillance) are freely available to other government agencies and non-profit organizations through a relatively straight-forward material transfer agreement. The database structure is also freely available through the Walter Reed Army Institute of Research’s Business and Agreements Office.

In addition to the above isolates, the following have also been sequenced as part of separate and/or ongoing investigations: ~1200 *Acinetobacter baumannii* isolated along the primary casualty evacuation route from theater to the two main military referral centers in the National Capital Region between 2003 and 2014 (of these approximately 87% carried *bla*_OXA-23_); 15 colistin-resistant *A*. *baumannii*; 20 extremely drug resistant (XDR)-highly virulent *A*. *baumannii* (fatal outbreak), 400 *Staphylococcus* spp, 200 MDR-*E*. *coli*; 100 MDR- or tigecycline resistant *Klebsiella*, 50 *C*. *difficile*, 10 vancomycin-resistant Enterbactereiacea (VRE); 10 drug-resistant *Salmonella/Shigella*, and 80 bacteriophage.

## Discussion

While implementing NGS across a large and geographically dispersed health system and surveillance network, we encountered several limitations, including lack of standardized quality control metrics, non-agile contracting and acquisition processes which cannot keep pace with technological improvements, limitations of short-read sequencing for investigating plasmid-based transmission dynamics, and the current limited availability of ultra-long read platforms such as the PacBio RS II or Sequel Sequencers (Table 2). Additionally, these occured in a setting of rapid and frequent and technological advances, and increasing regulatory and internet security requirements. Other larger organizations are likely to face these considerations and challenges as they implement enterprise-wide NGS-based surveillance. If these challenges are not addressed, they will likely constrain the broader application of this technology (especially at federal and state public health laboratories, or large health networks) for such pathogens [10]. Possible solutions or mitigating strategies are also listed in Table 2.

**Table 2:** Considerations and Challenges of Implementing Next Generation Sequencing Across Large Health Systems.

Consideration or Challenge	Possible Mitigation or Solution
**Data Generation**	
If pre-selecting isolates for NGS workflow basedon carbapenemnase production, the CarbaNP test may miss OXA-like carbapenemases in *Acinetobacter*	Do not use a negative CarbaNP to downselect *Acinetobacter* spp.; test with PCR or perform NGS on all isolates
Lengthy approval processes and laborious acquisition requirements; contract awards unable to keep pace with technologic advances	Allow cooperative research agreements with operations and maintenance type of funds; employ experienced acquisitions personnel within group to work closely with contracting agency; leverage flexible or agile contracting vehicles; vendors should notify contracting officer representatives or technical supervisors of impending major advancements or new releases; allow clinical operations to be funded with research and development monies (not solely operations and maintenance monies)
Balancing number of full time staff to workload	3–4 full time molecular laboratory technologists and one PhD level team lead for every 300–400 isolates sequenced per month
Limitations of shorter read platforms for certain types of bacterial antimicrobial resistance investigations (mobile genetic elements)	Increase access to or funding for positioning of ultra or very long read sequencing platforms at surveillance or referral laboratories
Limited availability of long read single molecule platforms	Wait for technologic advances to eliminate this constraint by making those platforms smaller and less expensive.
Compared to research laboratories, clinical laboratories are more susceptible to higher staff turnover and may not have staff with specialized training needed for preparing high quality DNA libraries	Increase and incentivize educational and training opportunities; leverage automation or robotics for library preparation
**Data Analysis**	
Balancing number of full time staff to workload	5–7 full time bioinformatacists and one PhD level team lead for every 300–400 isolates sequenced per month
Limited access to open source and other state of the art analytic software (primarily applies to government and military organizations)	Relax .mil restrictions on computer networks for facilities involved in biomedical research and clinical support; allow use of .org or .net; expedite process and shorten approval time for obtaining Certificates of .net Worthiness
**Data Sharing & Storage**	
Continuous sequencing of large volumes isolates (300–400 month) of creates extraordinary burdens for sharing and storage (Petabytes over the program lifecycle)	Increase bandwidth or provide infrastructure to accommodate emailing of FASTQ / FASTA data files of 10s to 100s of isolates at once; use tiered storage; explore vendor or cloud-based solutions (but these can be prohibitively expensive for larger projects)
Commercial 'off-the-shelf' database for managing isolate inventory and linking clinical and antibiotic susceptibility data to sequenced genomes to does not yet exist	Adopt the structure architecture of ARMoR-D which DOD can provide at no cost to nonprofit or other government agencies

ARMoR = Antimicrobial Resistance Monitoring and Research Program; CDC = U.S. Centers for Disease Control and Prevention; DOD = U.S. Department of Defense; NIH = U.S. National Institute of Health; FASTA/FASTQ = file format names for sequencing data

### Generating Data

NGS platforms differ in their sequencing chemistries, read-lengths, and throughput. Not all perform comparably for MDR-bacteria. Lefterova et al. offer a helpful review of platform types and specifications, along with specific applications to clinical bacteriology [17]. Very briefly, hydrogen ion detection (Ion Torrent) and reversible terminator (Illumina) platforms are based on ‘sequencing by synthesis’ (SBS) and use bridge amplification to amplify a single molecule into a cluster. The cluster contains ~1000 copies of the original molecule. All 1000 copies are sequenced together, one base at a time. Occasionally, the chemistry becomes dis-synchronous—as one of the 1000 copies may accidentally have two bases incorporated in a single cycle instead of just one. With repeated cycles, the dis-synchrony amplifies into what is referred to as ‘phase’ error. This is the primary error mode of amplified SBS chemistry and the main limit of achieving very long read lengths.

We found that hydrogen ion detection platforms did not provide the quality of sequence or read-length needed for many of our outbreak investigations of MDR-bacteria. Reversible terminator-based platforms provided better sequence quality and read length, but increasingly we found that the read lengths of that platform insufficient for: a) describing novel resistance mechanisms (i.e. an insertion element inside a promoter or gene amplification); b) closing certain bacterial genomes, and describing novel plasmids or species; c) determining transmission patterns involving small mobile genetic elements); and d) vetting or confirming certain results through peer-reviewed publication.

Single molecule technologies like PacBio and Oxford Nanopore are not affected by phasing error resulting in the generation of very long reads (generally limited only by the sample preparation process). The larger reads are easier to assemble due to more overlap between reads. Importantly, they also span repeats in the DNA sequence, allowing unambiguous location of the read on a reference, or use overlaps between reads to reconstruct the original DNA sequence. However, the cost and logistical support required for the ultra-long read / single molecule real time sequencing are daunting for many research laboratories, and prohibitive for most clinical laboratories. Less technically and logistically demanding, and less expensive alternatives are needed. Alternatively, greater access to and shorter turnaround times from the facilities that have this capability would be helpful. (During this study, we contacted all the facilities in the eastern half of the U.S. that had this technology, including the National Institute of Health and the Centers for Disease Control and Prevention, but none were able to assist us).

Another consideration relevant to generating data is purchasing supplies and equipment with public / government funding- especially the with the category of operations and maintenance funds that most clinical or hospital laboratories are funded with, which have to be spent in a single fiscal year (FY). This precludes the use of flexible or agile procurement vehicles, such as cooperative agreements, and also mandates that all funds must be obligated before the end of the FY. This has downstream effects such as being required to accept a years’ worth of consumables (space), or being unable to purchase supplies during the period between the end of the FY and the arrival of next FY funds. This limitation, in conjunction with abiding by existing federal acquisitions laws, results in lengthy pre-approval processes prior to contract award. The procurement process is not aligned to keep up with scientific innovation: on several occasions, better performing and less expensive platforms or kits were released shortly after the contract was awarded, making it too late for a course correction. For example, immediately after a government contract was announced for an RSII sequencer, the vendor announced the release of a much improved, smaller, less expensive version (Sequel), but was unwilling to re-negotiate. To this end, vendors should alert contracting officers and their representatives to impending major advances, and be more flexible. Governments should allow more agile procurement options and/or consider revising federal acquisition regulations for bio-medical efforts.

### Analyzing Data

Even with increasing automation, a multidisciplinary team of dedicated, highly trained, and experienced molecular microbiologists, laboratory technologists, bioinformaticians, and clinicians remain the most valuable asset for transforming the genomic data into biological and clinically useful knowledge. We suggest the creation of a number of fulltime bench and bioinformatic positions in order to consistently offer an analytic turnaround time of 48–72 hours for prioritized outbreak investigations, while routinely sequencing 300–400 isolates per month (Table 2).

Bioinformatic and information management remains one of the greatest challenges for implementing NGS across healthcare organizations or networks [7, 18]. Some of the best and most useful software applications are open source, and as such they require lengthy approval processes before installation on secure government computer networks (Table 1). Both http://www.mil-oss.org/ and http://www.forge.mil/, collaborative environments for civilian and military open source software and hardware developers, have been suggested as alternatives or solutions to the lengthy governances and the highly restrictive.mil environment [19]. However, they are neither feasible nor useful, especially for scientific and genomic research. Currently only very basic project tracking application are posted / available at both locations. Additionally, the time it takes for newly developed software to appear and be available on the site is even longer than obtaining individual governances and approvals.

### Sharing Data

**Fig 2:**
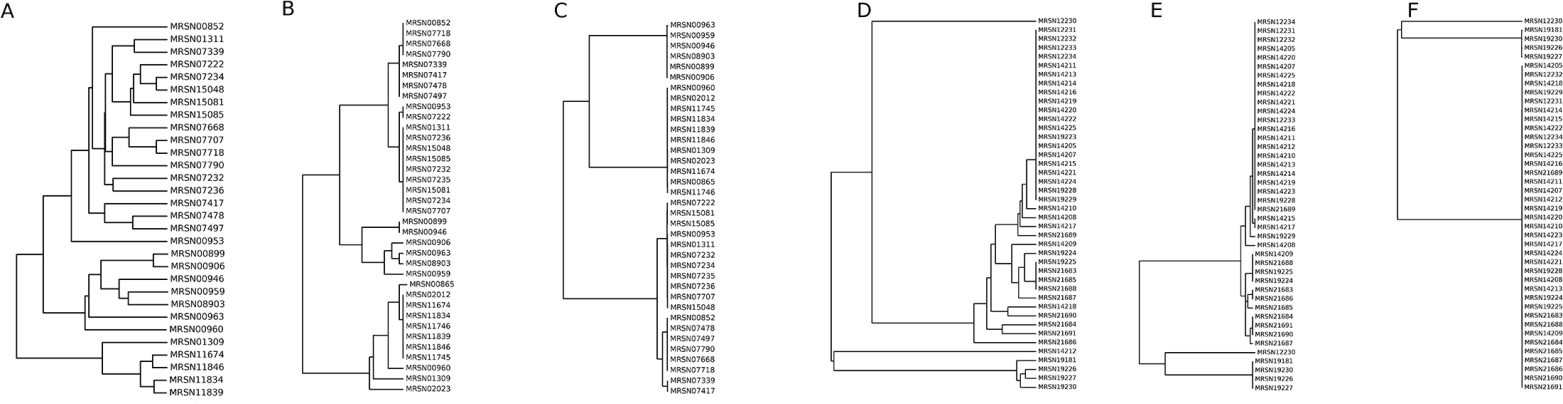
Phylogeny of Carbapenemase-producing Klebsiella and Carbapenemase-producing Acinetobacter. A-C represent the same *Klebsiella* isolates from the same outbreak event, and Fig 2D–2F represent the same *Acinetobacter* isolates from a separate same outbreak event. However, depending on the clustering method and software program used, different results can be obtained. This highlights the need for standardized approaches.

Insufficient bandwidth, lack of standardized sample preparation and analysis protocols, and definitions of NGS-based clonality limit the ability to send large data files or compare results between laboratories. For example, Fig 2A–2C depicts the same *bla*_oxa23_ carrying *Acinetobacter* isolates from the same outbreak event clustered by three equally valid methods—Genious, SNP and PFGE. Fig 2A–2C depicts the same *bla*_kpc_-carrying *Klebsiella* from the same outbreak event similarly clustered. Different conclusions regarding relatedness can be reached for the same outbreak event depending on which software was used, highlighting the need for standardized definitions. Further complicating this are the “challenge of the supragenome” and the high degree of genome plasticity. Most bacterial pathogens have a three component genome (core genes, distributed genes, and unique genes). The relative content and combinations of each change often- even *in situ* during a single chronic infection event [20]. This inherently affects clonality/relatedness. An outbreak can be analyzed and clustered based on either the core or the accessory genomes, or both.

Having laboratories provide raw-sequence data files to public databases (e.g. NCBI’s Genome Tracker) would facilitate standardized comparisons, but this is currently limited by secure file transfer protocols and bandwidth. Raw data files are very large and not amenable to emailing as attachments. Even for small projects, DVDs and external hard drives are currently the only available option (albeit insecure and inefficient) for transferring sequence data.

Reference materials for both proficiency testing and performing quality control facilitate laboratory accreditation and enhance comparability of results regionally, nationally, and internationally. To that end, we concur with others who advocate participation in the standardization and accreditation working groups [17, 21–23].

Other organizations have called for NGS standards, quality controls, and the availability of reference materials for proficiency testing [17, 21, 23], and we concur. To that end, our goal is to have our sequencing platforms accredited by the College of American Pathologists in 2017. In addition to achieving CAP accreditation for NGS, we will also participate in the Global Microbial Identifier's 2016 Proficiency Test to validate our processes and support the GMI Initiative [23].

### Storing Data

Inexpensive and cloud-based options may make data storage seem inconsequential. However, referral laboratories or those sequencing isolates from numerous facilities can quickly accumulate terabytes of data, and over the typical 5–10 program lifecycle, hundreds or thousands of terabytes. Sequencing 300–400 isolates/month, we generate ~3–6 Tb monthly.

### Managing Expectations

Although some of the problems discussed above are pathogen or government-peculiar and require similarly specific solutions, many are not. By keeping in mind the above considerations and challenges, clinicians, program managers, and other stakeholders can facilitate large-scale implementation of NGS, minimize delays, and maximize the chance of matching customer and stake-holder expectations with similar results and outcomes. Additionally, as ransomware attacks on healthcare organizations increase, [24] healthcare systems may choose or be forced to implement very tight internet and information management restrictions. Civilian organizations would then face the same IM challenges or constraints government and military networks face.

In summary, we use events encountered during the system-wide NGS-characterization of a broad collection of carbapenemase-producing bacteria to illustrate the considerations and challenges of large scale implementation of that technology to help others avoid potential difficulties, pitfalls and unmet expectations.

## References

[1] Centers for Disease Control. Antibiotic resistance threats in the United States, 2013. Available: http://www.cdc.gov/drugresistance/pdf/ar-threats-2013-508.pdf. Accessed 26 September 2015.

[2] Hayden MK. Measuring carbapenem-resistant Enterobacteriaceae in the United States: a critical step for control. JAMA. 2015; 314: 1455–1456. 10.1001/jama.2015.11629 26436518

[3] National Action Plan for Combating Antibiotic-resistant Bacterial. Available: https://www.whitehouse.gov/sites/default/files/docs/national_action_plan_for_combating_antibotic-resistant_bacteria.pdf. Accessed 5 November 2015.

[4] Manges AR. Genomic epidemiology: revealing hidden reservoirs for Klebsiella pneumonia. Clin Infect Dis. 2015;61: 900–902. 10.1093/cid/civ433 26206846 PMC4551006

[5] Lesho E, Waterman P, Chukwuma U, McAuliffe K, Neumann C, Julius MD, et al The Antimicrobial Resistance Monitoring and Research (ARMoR) Program: the Department of Defense’s response to escalating antimicrobial resistance. Clin Infect Dis. 2014;59: 390–397. 10.1093/cid/ciu319 24795331

[6] Davis RJ, Jensen SO, Van Hal S, Espedido B, Gordon A, Farhat R, et al Whole genomic sequencing in real-time investigation and management of a Pseudomonas aeruginosa outbreak on a neonatal intensive car unit. Infect Control Hosp Epidemiol. 2015;36: 1058–1064. 10.1017/ice.2015.133 26050101

[7] Fricke WF, Rasko DA. Bacterial genome sequencing in the clinic: bioinformatic challenges and solutions. Nature Rev 2014;15:49–55. 10.1038/nrg3624 24281148

[8] Hasman H, Saputra D, Sicheritz-Ponten S, Lund O, Svendsen CA, Frimodt-Møller N, et al Rapid whole-genome sequencing for detection and characterization of microorganisms directly from clinical samples. J Clin Microbiol 2014;52: 139–146. 10.1128/JCM.02452-13 24172157 PMC3911411

[9] Nature Publishing Group. Outbreak Genomics. Nature Biotechnology. 2011;29: 769 10.1038/nbt.1978 21904301

[10] Lesho E. How next generation sequencing might not transform infectious disease practice. Clin Infect Dis. 2016. In Press. 10.1093/cid/ciw008 26908790

[11] McGann P, Bunin JL, Snesrud E, et al Real time application of whole genome sequencing for outbreak investigation—what is an achievable turnaround time? Diag Microbiol Infect Dis. 2015. In press. 10.1016/j.diagmicrobio.2016.04.020 27185645

[12] Kong Y. Btrim: A fast, lightweight adapter and quality trimming program for next-generation sequencing technologies. Genomics 2011;98: 152–153. 10.1016/j.ygeno.2011.05.009 21651976

[13] Magoc T, Salzberg S. FLASH: Fast length adjustment of short reads to improve genome assemblies. Bioinformatics. 2011;27: 2957–2963. 10.1093/bioinformatics/btr507 21903629 PMC3198573

[14] Langmead B, Trapnell C, Pop M, Salzberg SL. Ultrafast and memory-efficient alignment of short DNA sequences to the human genome. Genome Biol. 2009;10: R25 10.1186/gb-2009-10-3-r25 19261174 PMC2690996

[15] Li H, Handsaker B, Wysoker A, et al The Sequence Alignment/Map format and SAMtools. Bioinformatics. 2009; 25: 2078–2079. 10.1093/bioinformatics/btp352 19505943 PMC2723002

[16] Zankari E, Hasman H, Cosentino S, et al Identification of acquired antimicrobial resistance genes. J Antimicrob Chemother. 2012;67: 2640–2644. 10.1093/jac/dks261 22782487 PMC3468078

[17] Lefterova MI, Suarez CJ, Banaei N, Pinsky BA. Next-generation sequencing for infectious disease diagnosis and management. J Mol Diag. 2015;17: 1–12. 10.1016/j.jmoldx.2015.07.004 26433313

[18] Koeser CU, Ellington MJ, Peacock SJ. Whole-genome sequencing to control antimicrobial resistance. Trends Genetics. 2014;30: 401–407. 10.1016/j.tig.2014.07.003 PMC4156311 25096945

[19] Pak TR, Kasarskis A. Reply to Lesho. Clin Infect Dis. 2016;62:1053. 10.1093/cid/ciw010 26908789

[20] Ehrlich GD, Post C. The time is now for gene and genome-based bacterial diagnostics. JAMA Intern Med. 2013;173: 1405–1406. 23857368 10.1001/jamainternmed.2013.7042

[21] Gargis AS, Kabman L, Berry MW, Bick DP, Dimmock DP, Hambuch T, et al Assuring the quality of next-generation sequencing in clinical laboratory practice. Nature Biotech. 2012;30: 1033–1036. 10.1038/nbt.2403 PMC3827024 23138292

[22] Aziz N, Zhao Q, Bry L, Driscoll DK, Funke B, Gibson JS, et al College of American Pathologists’ laboratory standards for next-generation sequencing clinical tests. Arch Pathol Lab Med. 2015;139: 481–493. 10.5858/arpa.2014-0250-CP 25152313

[23] Global Microbial Identifier. Available: http://www.globalmicrobialidentifier.org/Workgroups/About-the-GMI-Proficiency-Test-2015. Accessed 5 November 2015.

[24] US News & World Report: A Health Hack Wakeup Call. Available: http://www.usnews.com/opinion/blogs/policy-dose/articles/2016-04-01/ransomware-hacks-are-a-hospital-health-it-wake-up-call. Accessed 3 April 2016.

